# Electrospun Metal–Organic Framework-Fabric Nanocomposites as Efficient Bactericides

**DOI:** 10.1021/acs.langmuir.3c01039

**Published:** 2023-06-29

**Authors:** Mohammad H. Hashem, Mohamad Wehbe, Patrick Damacet, Rayan Kadah El Habbal, Nesreen Ghaddar, Kamel Ghali, Mohammad N. Ahmad, Pierre Karam, Mohamad Hmadeh

**Affiliations:** † Department of Mechanical Engineering, American University of Beirut, Beirut 1107 2020, Lebanon; ‡ Chemistry Department, American University of Beirut, P.O. Box 11-0236, Riad El-Solh, 1107 2020 Beirut, Lebanon; § Bahaa and Walid Bassatne Department of Chemical Engineering and Advanced Energy, Faculty of Engineering and Architecture, American University of Beirut, P.O. Box 11-0236, Beirut 1107 2020, Lebanon

## Abstract

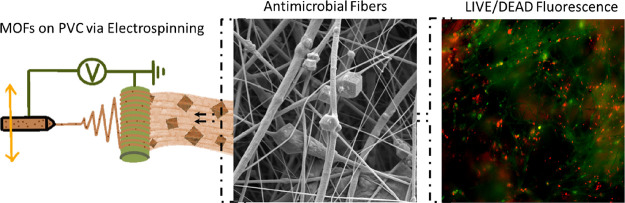

In this work, we utilized electrospinning to develop advanced composite membranes of polyvinyl chloride (PVC) loaded with postmetalated metal–organic frameworks (MOFs), specifically UiO-66(COOH)_2_-Ag and ZIF-8-Ag. This innovative technique led to the creation of highly stable PVC/MOFs-Ag membrane composites, which were thoroughly characterized using various analytical techniques, including scanning electron microscopy, powder X-ray diffraction, thermogravimetric analysis, X-ray photoelectron spectroscopy, porosity analysis, and water contact angle measurement. The results verified the successful integration of MOF crystals within the nanofibrous PVC membranes. The obtained composites exhibited larger fiber diameters for 5 and 10% MOF loadings and a smaller diameter for 20% loading. Additionally, they displayed greater average pore sizes than traditional PVC membranes across most MOF loading percentages. Furthermore, we examined the antibacterial properties of the fabricated membranes at different MOFs-Ag loadings. The findings revealed that the membranes demonstrated significant antibacterial activity up to 95% against both Gram-negative (*Escherichia coli*) and Gram-positive (*Staphylococcus aureus*) bacteria as the MOFs-Ag loading increased, even when maintaining a constant silver concentration. This indicates a contact-based inhibition mechanism. The outcomes of this study have crucial implications for the development of novel, stable, and highly effective antibacterial materials, which could serve as superior alternatives for face masks and be integrated into materials requiring regular decontamination, as well as potential water filtration systems.

## Introduction

Outbreaks of communicable diseases have existed ever since the hunter-gatherer days of humankind. Microorganisms like bacteria, viruses, fungi, and parasites are the major causes of infectious diseases in humans as they lead to nearly 1.5 billion total disability-adjusted life years.^1^ Moreover, the continuous evolution of these microbial organisms due to mutations and strain reassortment has made some vaccines and antibiotics limited in their use.^2^ The effects of outbreaks such as that of the COVID-19 pandemic on the health of every individual worldwide are nothing short of devastating.^3^ As a response to such outbreaks, there is a pressing need to search for new ways not only to protect humans from airborne pathogens that are highly transmissible but also to develop protective technologies that can act as a safeguard against diseases that are transmitted by contaminated textile materials and surfaces.^4−7^ The most rapid response in defense to airborne pathogens is the usage of personal protective equipment (PPE), especially surgical face masks, to limit the means of infection.^5,8−10^ Various strategies have been previously explored to increase the durability of face masks, their reusability, and their antimicrobial activity by using different polymers and incorporating different biocidal agents like Ag nanoparticles, Cu nanoparticles, or reactive oxygen species.^3^ Metal–organic frameworks (MOFs) are a hybrid of organic–inorganic porous crystalline materials made up of metal clusters bridged together by organic linkers.^11^ Because of their ideal textural properties, high surface area, porosity, and the ease of their modification with bactericidal components such as reactive oxygen species generating materials and silver nanoparticles, MOFs have emerged as a promising material to be incorporated in PPE, especially surgical face masks.^12−14^ Different variations of MOFs such as zeolitic imidazolate framework-8 (ZIF-8) have been incorporated into fibrous membranes for enhancing filtration.^15^ These face masks have shown to have antibacterial activity against *Staphylococcus aureus* and are efficient in protecting against COVID-19.^16^ Limiting disease spread through contaminated surfaces has also been addressed by the usage of MOFs as a self-cleaning photoactive material that prevents contamination of clothing. Of interest is the usage of MOFs in the prevention of bacterial biofilm formation. Hospital-acquired infections account for 1.7 million infections each year, half of which are attributed to the growth of bacterial biofilms.^17−19^ These infections usually occur due to the colonization of bacteria on medical device surfaces, which indicates the need for new bactericidal techniques.^19,20^

Silver has been known for its effectiveness against a wide range of microbes including Gram-negative and Gram-positive bacteria.^21,22^ Although the antibacterial mechanism of silver is still yet to be entirely clarified,^23^ various mechanisms of action have been proposed. Indeed, silver ions can disrupt bacterial cell walls and cytoplasmic membranes, denature bacterial ribosomes, interfere in bacterial DNA replication, and perforate and disrupt membranes by the generation of reactive oxygen species.^24,25^ Although silver has gained a lot of popularity in the biomedical sciences, its controlled release in concentrations that are safe for humans remains an area of extensive research.

Recently, electrospinning (ES) has gained a lot of recognition because of its ability to be used in synthesizing polymeric nanofibrous materials through the application of a high electrical voltage on a polymer solution.^26^ Through this process, it is possible to synthesize nanofibers of various forms and sizes such as membranes, which have the potential to be incorporated as bactericidal tools for medical devices and face masks or simply used to prevent surface contamination by inhibiting bacterial biofilm formation.^27^ Electrospun nanofiber membranes are promising polymer composites that can be used as textile fabric surfaces. Because of their small pore size that allows filtration of particles and infectious agents, their ability to be reused when disinfected, and their big surface area and flexibility,^26,28^ these membranes have the potential to be employed in unique surface decontamination methods to inhibit biofilm formation and potentially present a better alternative to face masks. Previously, our group developed new antimicrobial agents based on silver postmetalated-zirconium-based MOF crystals that exhibited a good potency with a calculated minimum inhibitory concentration (MIC) and minimum bactericidal concentration (MBC) of 6.5 μg/mL silver content.^13^

Herein, this work aims to develop a new prototype of antibacterial films by synthesizing highly stable and reusable electrospun polyvinyl chloride (PVC) membranes combined with the long-term persistence and thermal and optical stability of UiO-66(COOH)_2_ and ZIF-8 postmetalated with silver. As a proof of concept, the bactericidal activity of these membranes is evaluated against *Escherichia coli* and *S. aureus* in which the membranes showed good potency and bacterial inhibitory properties.

## Experimental Section

### Materials and Chemicals

All chemical reagents and solvents used throughout this work were commercially supplied and utilized without further purification*. N*,*N*-Dimethylformamide (DMF) (purity ≥99.8%), tetrahydrofuran (THF) with ≥99.8% purity, methanol (ACS reagent, >99,8%), PVC with an average molecular weight of 80,000 g/mol and a density of 1.4 g/mL at 25 °C, solid silver nitrate (AgNO_3_, 99.8%), zirconyl chloride octahydrate (ZrOCl_2_·8H_2_O), zinc chloride (ZnCl_2_), 1,2,4,5-benzenetetracarboxylic acid (C_10_H_6_O_8_), 2-methylimidazole (C_4_H_6_N_2_), formic acid (ACS reagent, 88–91%), and Luria-Bertani (LB) broth were all purchased from Sigma-Aldrich. Mueller-Hinton broth (MHB) was acquired from Fisher Scientific. Commercial polyester screen fabric with an average pore diameter of 350 μm and a thickness of 215 ± 2 μm was used to cover the cylindrical collector (*D* = 10 cm) of the ES machine.

### MOF Preparation

#### Synthesis of UiO-66(COOH)_2_

UiO-66(COOH)_2_ was prepared via a solvothermal method by a synthesis route previously reported in the literature.^13^ Briefly, an equimolar amount of zirconyl chloride octahydrate (0.185 mmol, 59.6 mg) and 1,2,4,5-benzenetetracarboxylic acid (0.185 mmol, 47.1 mg) were dissolved in a 20 mL scintillation vial containing 4 mL of DMF and 4 mL of formic acid modulator. The mixture was then homogenized by sonication and placed in a preheated oven for 5 h at a temperature of 130 °C. The resulting white UiO-66(COOH)_2_ particles were subjected to an extensive washing process for proper crystal activation. They were first washed by soaking them in DMF, which was changed at least three times a day for three consecutive days. DMF was then exchanged by methanol and the crystals were washed three times for another 3 days to ensure that no DMF remained in the pores. Finally, the MOF crystals were dried under dynamic vacuum for 12 h at 80 °C for complete pore evacuation.

#### Synthesis of UiO-66(COOH)_2_-Ag

The prepared UiO-66(COOH)_2_ (20 mg) was added to a silver solution containing 40 mg of silver nitrate (AgNO_3_) dissolved in 10 mL of methanol. The mixture was then stirred and heated on a hot plate for 20 h at a temperature of 50 °C to allow for the incorporation of silver into the framework. The resulting brown crystalline powder was collected by centrifugation, washed with DMF and methanol for two consecutive days each, and finally dried under vacuum at 80 °C for 12 h.^13^

#### Synthesis of ZIF-8

ZIF-8 was prepared via a reaction–diffusion process by diffusion of a 2-methylimidazolate-based solution into an agar gel matrix containing zinc metal cations.^29^ In brief, the inner portion was first prepared by dissolving 136 mg of zinc chloride (50 mM) in a 1:1 mixture of DMF and water, followed by the addition of 1% (w/w) bacteriological agar powder to the mixture. The resulting solution was then heated and stirred on a hot plate to allow for the complete dissolution of the agar gel which was then transferred to a Pyrex tube filling it to two-thirds. After complete gelation of the agar gel, the outer portion was prepared by dissolving 247 mg of 2-methylimidazolate (500 mM) in a 1:1 mixture of water and DMF. The resulting solution was then poured on top of the inner electrolyte filling the rest of the Pyrex tube, covered with parafilm, and left on the bench for a few days to allow for the reaction–diffusion process and formation of the white precipitate front to take place. The precipitation regions of ZIF-8 were thereafter extracted. Washing and activation procedures were similar to those of UiO-66(COOH)_2_. The crystals were washed with DMF to dissolve the agar gel and later activated by solvent exchange with methanol. Finally, the particles were collected by centrifugation and dried under vacuum for 12 h at 100 °C.

#### Synthesis of ZIF-8-Ag

ZIF-8-Ag was prepared by introducing silver precursors to ZIF-8, followed by reduction.^30^ In brief, 100 mg of ZIF-8 was added to a silver solution containing 17 mg of silver nitrate (AgNO_3_) dissolved in 4 mL of DI water. The resulting mixture was first sonicated and later stirred on a hot plate for 12 h at room temperature. The ZIF-8 suspension was then collected by centrifugation and dried under dynamic vacuum at 80 °C for 12 h. The light-gray crystalline powder was thereafter soaked in a water solution containing 50 mg of the NaBH_4_ reducing agent. The mixture was then stirred for 30 min, resulting in the formation of a dark gray powder corresponding to the ZIF-8-Ag. Finally, the resulting product was washed with methanol, collected via centrifugation, and dried under vacuum for 12 h at 80 °C.

### Polymeric Solution Preparation

PVC solution (16 wt %) was prepared by adding 2.64 g of PVC powder to a 15 mL mixture of DMF:THF 10:5 (v/v). The mixture was stirred at room temperature for about 12 h at 600 rpm for complete dissolution. Before ES, the polymeric solution was allowed to rest to remove entrapped air bubbles.

As for preparing PVC/MOFs-Ag solution, PVC was dissolved using 2/3 of solvent mixture. The other 1/3 was used to disperse the MOFs-Ag of different percentages (5, 10, and 20 wt %) of polymer using sonication for 30 min. Finally, the two solutions were mixed and stirred for another 30 min.

For comparative reasons, PVC/AgNO_3_ solutions were also prepared as control samples by dissolving the desired percentages (5, 10, and 20 wt %) of AgNO_3_ salt in 2 mL of the same organic solvent mixture and then adding it to the completely dissolved PVC solution.

### Nanofibrous Membrane Fabrication

Producing nanofibrous thermoplastic polymeric membranes using ES can be quite challenging, especially upon adding other materials to the main solution. Many parameters can play a key role in determining the morphology and the stability of production. The synthesis of these nanofibrous membranes relies on the solution concentration, viscosity, conductivity, type of solvent, and ES conditions, which include the applied voltage, pumping flow rate, and tip-to-collector distance.^31^

**Figure 1:**
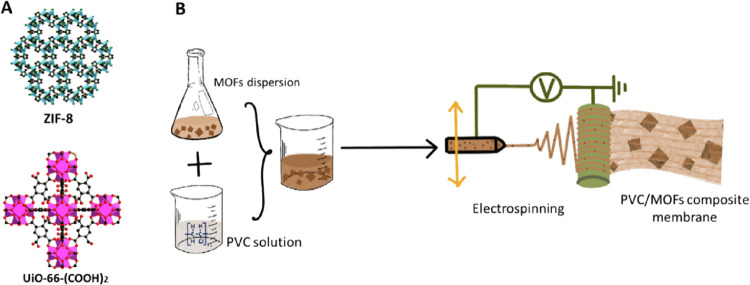
Crystal structures of ZIF-8 and UiO-66(COOH)_2_ used in this study (A). Schematic illustration of PVC/MOFs-Ag production via ES (B).

The membranes are fabricated using a lab-scale ES machine (Fluidnatek by Bioinicia). The drum collector (*D* = 10 cm) was first covered with a polyester fabric. A 20 mL plastic syringe was then filled with the polymeric solution, which was then fed to the nozzle at a flow rate of 3 mL/h using a syringe pump. It is important to note that the addition of either the UiO-66(COOH)_2_-Ag, ZIF-8-Ag, or AgNO_3_ to the main polymer solution changes its conductivity and viscosity. Therefore, the ES parameters must be modified to obtain the best stable polymer jet possible. The ES conditions (Table S1) used to produce the nanofibrous membranes were taken after a sequence of selection and optimization steps. In the case of PVC/ZIF-8-Ag, the same ES conditions as untreated PVC were employed without any changes. However, for the PVC/UiO-66(COOH)_2_-Ag and PVC/AgNO_3_, different voltages were used. All membranes were collected after 2.5 h of ES. Three membrane systems are synthesized in this work, PVC/ZIF-8-Ag (P-Z), PVC/UiO-66(COOH)_2_-Ag (P-U), and a reference membrane PVC/AgNO_3_ (P-A). Each system includes three different silver-metalated MOF loading percentages (5, 10, and 20%) in addition to the untreated PVC membrane, which adds up to 10 different membranes in total (Table S1). A schematic illustration of the process is shown in Figure 1. All membrane and MOF characterizations are described in the SI.

### Antibacterial Property Tests

The antibacterial activity of postmetalated MOFs on PVC surfaces was tested against Gram-negative *E. coli* (ATCC 25922) and Gram-positive *S. aureus*. The MIC, which describes the minimum concentration of silver needed to inhibit bacterial growth, and the MBC, which describes the concentration of silver needed to kill bacteria, against *E. coli* and *S. aureus* were calculated according to the Clinical & Laboratory Standards Institute (CLSI) protocols and as described in more detail by Wiegand et al.^24^ A few bacterial colonies (*E. coli* or *S. aureus*) were scraped from the surface of a freshly prepared plate and inoculated into 3 mL of LB broth solution and incubated overnight at 37 °C with constant shaking at 150 rpm. The overnight culture was then centrifuged, and the supernatant was then discarded. PBS solution (5 mL) was added to the formed pellet. The suspension was then vortexed and 100 μL was transferred to 3 mL of MHB and incubated for 3–4 h to achieve a solution of bacterial cells in the lag phase. The absorbance of the sample was assessed using a NanoDrop 2000c spectrophotometer (Thermo Fisher Scientific) and diluted to be in the range of the 0.5 McFarland standard (OD_600_ nm between 0.08 and 0.013). The obtained suspension was then diluted to reach a final bacterial concentration of 1 × 10^5^ and 1 × 10^6^ CFU/mL.

PVC containing different loading percentages of silver-metalated MOFs were then weighed, cut into small pieces using sterile forceps, and then sterilized by UV light under a biosafety cabinet hood for 15 min. The membranes were then suspended in MHB at a set concentration and then incubated with *E. coli* or *S. aureus* for 18 h at 37 °C with shaking at 200 rpm. The turbidity of the obtained samples was then assessed visually and the OD_600_ was measured using a NanoDrop 2000c spectrophotometer to determine the MICs of the samples. A brief illustration is shown in Figure S1. The percentage bacterial inhibition was calculated through the formula of eq 1:
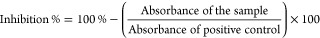
1

For comparison purposes, we will be reporting an effective inhibition factor (EI) calculated from the ratio of the inhibition percentage to the measured silver content in μg/mL, as shown in eq 2:
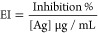
2

When comparing EIs of different samples, we still ensure that the silver concentrations are close in value.

As for the MBC, which is defined as the lowest concentration of silver that resulted in 99.9% of bacterial killing, it was determined by taking 100 μL of aliquots from the growth tubes and plating them on LB agar plates. The LB agar plates were then incubated at 37 °C for 18 h and then visually inspected for colony growth.

## Results and Discussion

### MOF Properties

**Figure 2:**
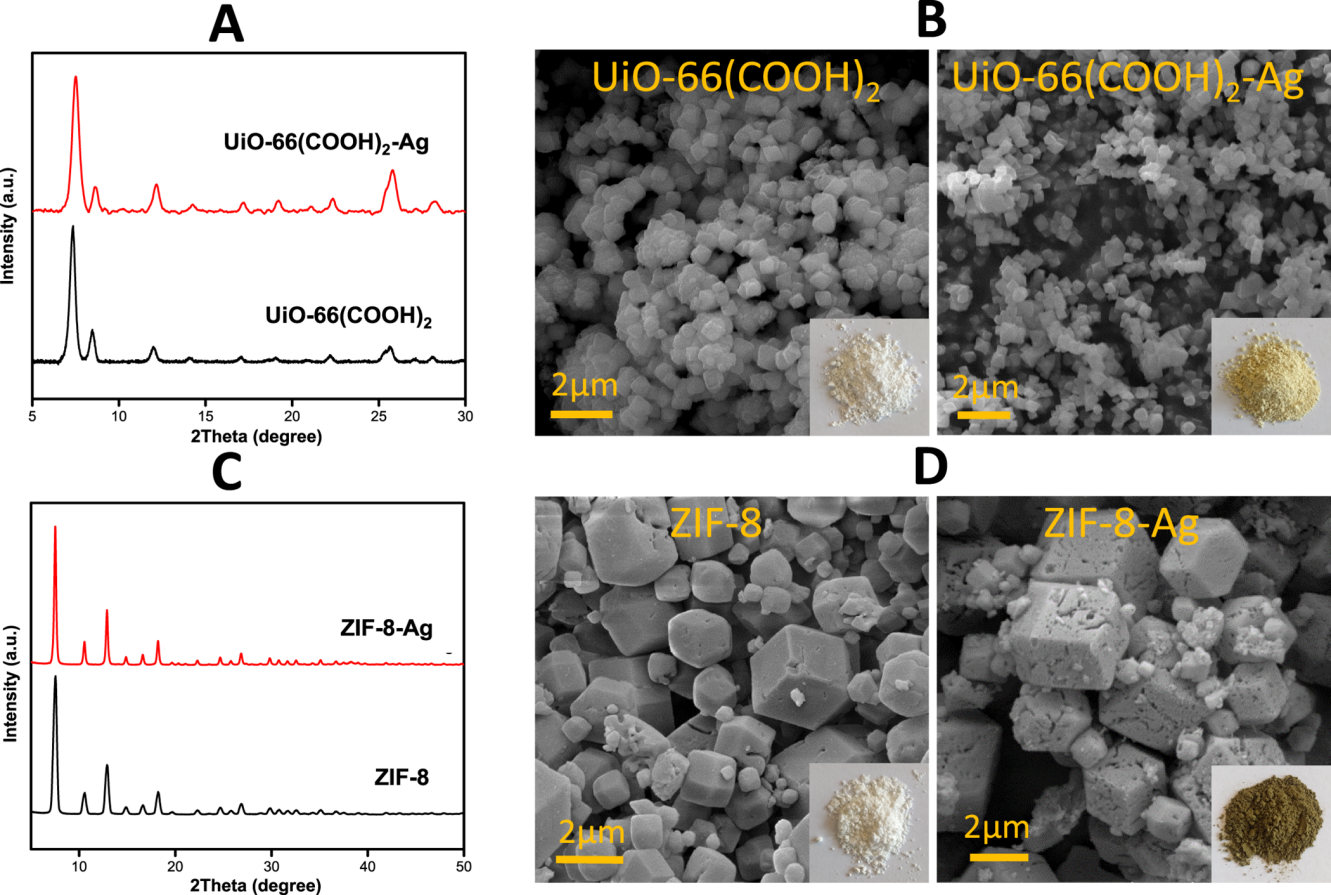
(A) PXRD pattern of UiO-66(COOH)_2_ (bottom-black) and UiO-66(COOH)_2_-Ag (top-red). (B) Optical and SEM images of UiO-66(COOH)_2_ and UiO-66(COOH)_2_-Ag. (C) PXRD pattern of ZIF-8 (bottom-black) and ZIF-8-Ag (top-red). (D) Optical and SEM images of ZIF-8 and ZIF-8-Ag.

The powder X-ray diffraction (PXRD) patterns of the MOFs and their metalated forms were well resolved and in good agreement with the simulated ones, confirming the high crystallinity and the phase purity of the produced samples (Figure 2A,C). Furthermore, no additional peaks could be observed for silver-based crystalline species due to the low loading and high dispersity on the UiO-66(COOH)_2_ and ZIF-8 crystals. As can be seen from the scanning electron microscopy (SEM) images (Figure 2B), the crystals of UiO-66(COOH)_2_ (white) and UiO-66(COOH)_2_-Ag (yellow) were of octahedral shapes with an average particle size of 0.5 ± 0.1 μm, while ZIF-8 (white) and ZIF-8-Ag (greenish-gray) samples exhibited a rhombic dodecahedron morphology and an average size of 3 ± 1 μm (Figure 2D). These data show that the crystalline nature and morphology of the studied MOF structures are preserved upon Ag metalation.

Thermogravimetric analysis (TGA) of the metalated and non-metalated MOF samples (Figure S2) showed that the weight % remaining in both metalated MOFs was higher than that of non-metalated ones. This is due to the presence of silver metal in the samples. The lower decomposition temperature of UiO-66(COOH)_2_-Ag (380 °C) compared to non-metalated UiO-66(COOH)_2_ (565 °C) suggests that the introduction of silver (Ag) into UiO-66(COOH)_2_ reduces its thermal stability. On the other hand, ZIF-8-Ag is more stable than ZIF-8 at temperatures below 480 °C and of comparable thermal stability at higher temperatures. This implies that while silver metalation decreased the thermal stability of UiO-66(COOH)_2_, it has increased it for ZIF-8. The silver content in the metalated MOFs was estimated using AAS and found to be 8.3 wt % for ZIF-8-Ag and 9.1 wt % for UiO-66(COOH)_2_-Ag.

To shed more light on the binding of Ag to the frameworks, the FTIR spectra were recorded for the MOFs and their postmetalated forms. The FTIR spectrum of UiO-66-(COOH)_2_ shows the presence of two bands at 750 and 1413 cm^–1^ corresponding to Zr–O and for COO– stretching vibrations, respectively (Figure S3). The band around 1700 cm^–1^ and O–H stretching vibrations at 2500–3100 cm^–1^ confirm the existence of non-coordinating carboxyl groups available for the Ag postmetalation.^13^ An absorption band within 3100–3500 cm^–1^ suggests the presence of O–H of −COOH or water molecules. The IR spectrum of UiO-66-(COOH)_2_-Ag indicates a decrease in O–H and free C=O stretching vibrations at 2500–3100 and 1700 cm^–1^, respectively, suggesting the coordination of free carboxyl groups with silver cations. As for ZIF-8, the peaks observed at 450 and 950–1200 cm^–1^ can be attributed to the stretching vibrations of Zn–N and C–N, respectively. The bands between 1500 and 1600 cm^–1^ correspond to phenyl ring vibrations. Moreover, the bands between 3135 and 2930 cm^–1^ were attributed to the aromatic and aliphatic C–H stretching of methylimidazole. The decrease in the C=N stretching peak at 1584 cm^–1^ in the ZIF-8-Ag spectrum indicates the interaction of silver with the ZIF framework (Figure S3).

**Figure 3:**
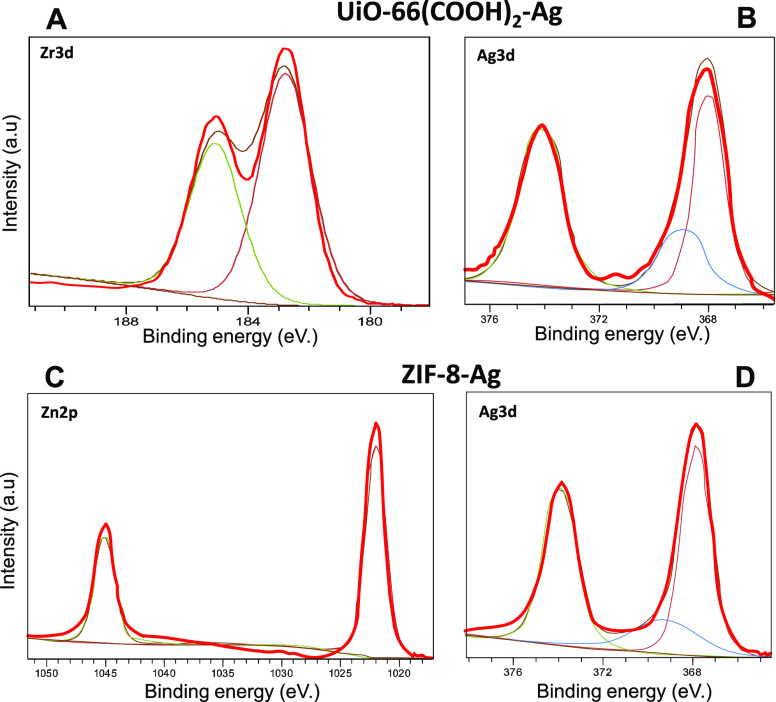
XPS spectra of Zr 3d and Ag 3d of UiO-66(COOH)_2_-Ag (A, B) and of Zn 2p and Ag 3d of ZIF-8-Ag, respectively (C, D).

To gain more insight on the oxidation states of Ag species, X-ray photoelectron spectroscopy (XPS) analysis was performed for UiO-66(COOH)_2_-Ag and ZIF-8-Ag. The XPS survey spectrum of the UiO-66(COOH)_2_-Ag sample showed the existence of Ag 3d_3/2_ and Ag 3d_5/2_ peaks at 368 and 374 eV, respectively, which indicates the successful incorporation of Ag into the UiO-66(COOH)_2_ structure. A deconvolution analysis of Ag 3d peaks allowed us to suggest the presence of two Ag species, Ag(0) and Ag^+^.^32^ In addition, Zr 3d peaks were observed in the binding energy range of 180–187 eV and can be divided into four peaks that correspond to Zr–O bonds (182.7 and 185.2 eV) and to Zr–Zr bonds (183.0 and 185.7 eV). A similar observation can be found in the XPS spectrum of the ZIF-8-Ag sample, which also suggests the existence of both Ag(0) and Ag^+^ species. Indeed, the Ag (3d_5/2_) binding energies of Ag(0) and Ag^+^ were located at 369.2 and 367.4 eV, respectively. As for the Zn 2p spectrum, the characteristic binding energies for Zn 2p peaks are found in the range of 1020–1025 eV for Zn 2p_3/2_ and 1040–1045 eV for Zn 2p_1/2_ (Figure 3).

### Membrane Characterization

**Figure 4:**
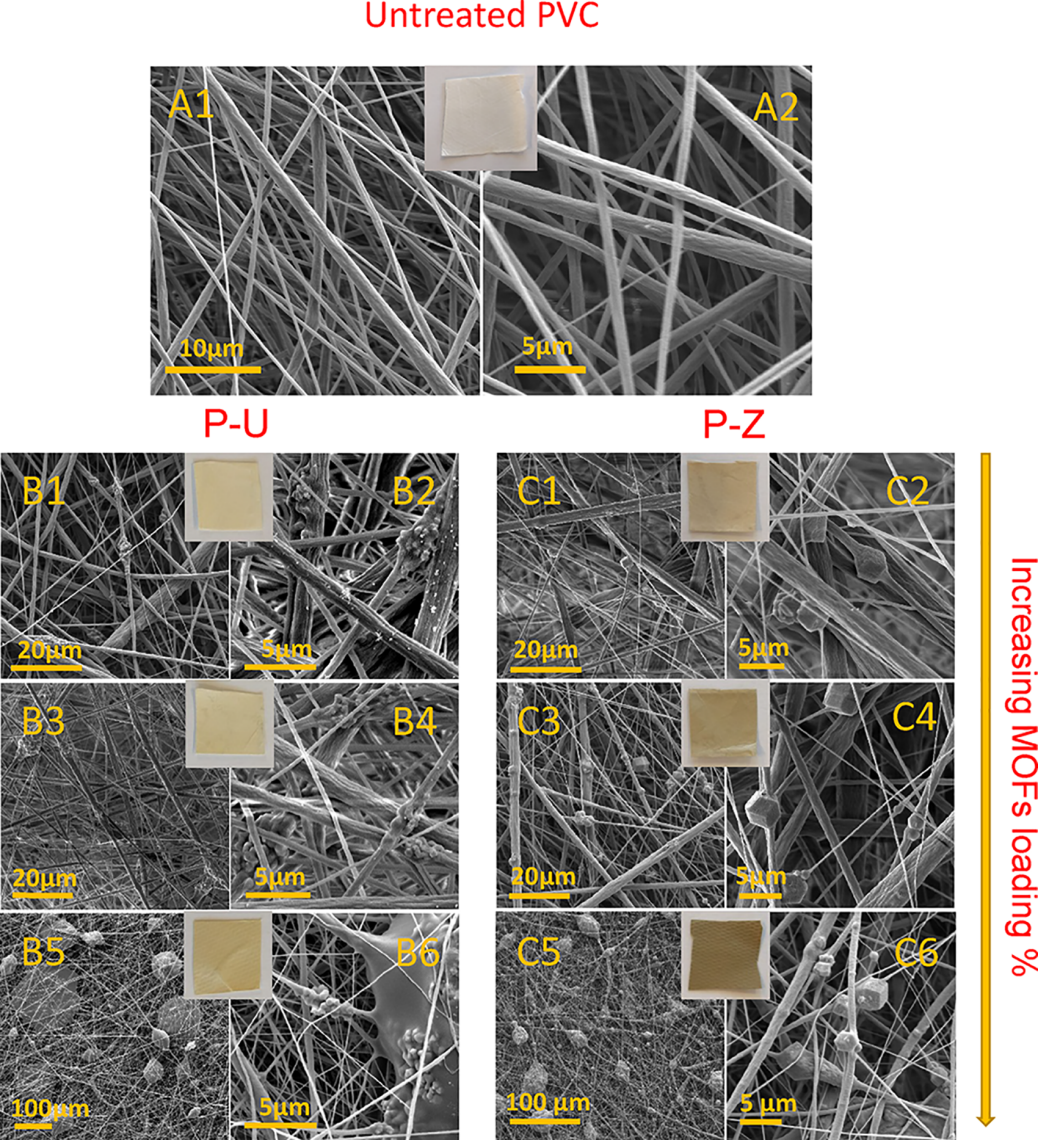
SEM and optical image (1 × 1 cm) of the untreated PVC membrane (A1, A2). PVC/UiO-66(COOH)_2_-Ag 5% (B1, B2), 10% (B3, B4), 20% (B5, B6); PVC/ZIF-8-Ag 5% (C1, C2),10% (C3, C4), 20% (C5, C6).

The optical and SEM images of the untreated PVC membranes produced by ES are shown in Figure 4A1,A2. These PVC membranes were white in color and displayed smooth continuous fibers with an average diameter of 0.7 ± 0.3 μm.

Upon addition of the small UiO-66(COOH)_2_-Ag crystals to the PVC solution, the morphology of the PVC/MOFs-Ag system (Figure 4) became distinguishable from that of pristine PVC as seen in the SEM images of the MOFs-Ag-loaded membrane (P-U). In fact, we can clearly visualize embedded MOFs-Ag inside the PVC fibers in the form of clumps and aggregates. The aggregates’ size increased to a point where the ES jet was unstable, and the membrane was full of bead-like structures with an average diameter of 15.5 ± 7.8 μm at 20% MOFs-Ag loading (Figure 4B5). In the case of 5 and 10% loading, the presence of the UiO- 66(COOH)_2_-Ag had a minor effect on the fiber diameter. This effect, however, was more significant at 20% loading as seen in Figure S4. This can be explained by the presence of a high amount of silver in the solution, which resulted in a higher solution conductivity and thus higher fiber stretching and a lower fiber diameter. Moreover, the presence of the UiO-66(COOH)_2_-Ag in the membrane changed its color from white to yellowish, which became darker at higher loading percentages (Figure 4). The TGA results of the untreated PVC nanofibrous membrane and the P-U samples are shown in Figure S5. We noticed that at 400 °C, the remaining weight percentage of untreated PVC was around 38% compared to 44% for P-U at 20% loading. This difference in the weight % loss is due to the presence of silver-metalated MOFs in the membrane.

Unlike P-U membranes, ZIF-8-Ag crystals in P-Z samples were mostly embedded inside the PVC fibers, as shown in the SEM images in Figure 4C1–C6. This is mainly due to the larger crystal size of the ZIF-8-Ag. At 5 and 10% loadings, the fiber diameter increased from 0.7 μm (for the untreated PVC) to 1.1 and 1 μm, respectively. In the case of 20% ZIF-8-Ag loading, however, a minor effect (0.7 ± 0.4 to 0.6 ± 0.6 μm) on the fiber diameter was observed (Figure S4). It was also observed that the amount of ZIF-8-Ag increased with increasing loading percentage until big bead-like structures were observed at 20% loading with an average diameter of 27.4 ± 4 μm (Figure 4C5). Such beads were not observed in the case of 5 and 10% loadings. The observed color of the membranes was greenish-gray, which became more prominent as the percentage (%) of Ag loading increased (Figure 4). Adding ZIF-8-Ag showed a significant impact on the treated membrane’s thermal behavior. As demonstrated in Figure S6, all ZIF-8-Ag-treated samples (P-Z) were less stable in terms of mass loss as they started a rapid sharp degradation at 240 °C compared to the untreated PVC, which showed a broad rapid degradation starting from 260 °C. Unlike the PVC membrane, which lost 62% of its original mass at 400 °C, P-Z membranes lost much less reaching around 45% in the case of 20% MOFs-Ag loading. In fact, untreated PVC was completely carbonized at around 600 °C, while P-Z (20%) was still holding 30% of its mass, which corresponds to the silver-metalated MOF species.

To appraise the role of the MOFs-Ag structure in the fabricated membranes, reference membranes incorporating only AgNO_3_ silver salts were synthesized under similar conditions to those employed for P-U and P-Z. SEM images of P-A membranes showed no noticeable effect on the morphology of membranes (Figure S7A1,A2) for 5% AgNO_3_ loading compared to the untreated PVC of Figure 4A1,A2. Meanwhile, for 10% (Figure S7A3,A4) and 20% (Figure S7A5,A6) MOFs-Ag loadings, it was clear from the SEM images that small spherical nanoparticles were located on the surface of the fibers with no change in their overall structure. One thing to note is that there is an insignificant decrease in the fiber diameter at 5 and 10% of the P-A membrane and a significant increase at 20% (Figure S4) when compared with the untreated PVC. The major change in the fiber diameter is related to the change in the conductivity of the solution after the addition of AgNO_3_ silver salt. This has been proven to affect the solution behavior in ES and consequently affect the fiber diameter. While the three prepared membranes were white at the beginning, they turned brown due to oxidation of the silver nitrate. The intensity of the color increased with the increase in the loading percentages (Figure S7). Also, while the addition of AgNO_3_ to PVC had a small impact on the thermal behavior of the membrane at low temperatures (<310 °C), it had a noticeable effect at higher temperatures where P-A membranes showed a rapid decrease in mass % starting at 550 °C. On the other hand, untreated PVC membranes showed a fast decrease at 440 °C, which continued to gradually decrease until the end. It is worth mentioning that at temperatures above 600 °C, all the membranes reached a final plateau with a higher percentage loading of the membrane leading to a greater mass retention after the decomposition process was over (Figure S8).

The porosity of nanofibrous membranes was usually determined by the fiber diameter. When particles were added to the membrane, the porosity and the overall physical and chemical properties of the membrane were affected by the amount and size of the added particles, which consequently altered the fiber diameter as well as the fiber stacking. In theory, there is a positive correlation between the fiber diameter and the pore size such that if the fiber diameter is small, the packing density would be higher and thus the pore size would be smaller.^33^ As can be seen in Figure S9, the untreated PVC membrane has an average and maximum pore size of 2.6 and 4.6 μm, respectively. With the addition of 5 and 10% AgNO_3_, these values remained nearly the same as that of the untreated PVC membrane. However, they were much higher for the 20% P-A. These results are consistent with the fiber diameter results shown above (Figure S4). As for P-U and P-Z, the pore size had noticeably changed. Low concentrations of MOFs-Ag (5%), namely, both ZIF-8-Ag and UiO-66(COOH)_2_-Ag, caused a significant increase in the average and maximum pore size when compared to the untreated PVC. P-U showed a decrease in the average and maximum pore size with the increasing MOFs-Ag loading % until it reached 0.5 and 4.3 μm, respectively, at 20% MOFs-Ag loading. This can be explained by the smaller fiber diameter obtained when MOFs-Ag particles coagulate at high loading percentages, leading to more stacking and packing density and thus, a smaller pore size, as was shown previously in the SEM images in Figure 4 and Figure S4. In the case of P-Z, the large crystal size of the MOFs prevented the fibers from agglomeration, which led to a decrease in fiber packing density and consequently an increase in the pore size. The further increase in loading % did not affect the pore size, even though the fiber diameter was relatively higher. This is related to the size of the ZIF-8 crystals, which showed minor changes compared to P-U. This indicates that the size of the MOFs-Ag had a significant effect on the pore size of the loaded membranes.

The FTIR spectrum of PVC exhibits characteristic peaks around 2850–3000 cm^–1^, which are associated with C–H and C–H_2_ stretching vibrations of the polymer backbone^34,35^ (Figure S10). The C–H aliphatic bending bond is designated to the peaks located at around 1400 cm^–1^, as well as peaks around 1000–800 cm^–1^ that are indicative of C–H bending vibrations. Peaks ranging between 600 and 650 cm^–1^ are related to the C–Cl bond.^35,36^ Similar peaks can be observed in the cases of P-U and P-Z. However, there is an additional characteristic peak in the FTIR spectrum of P-U at ≈1630 cm^–1^, which corresponds to the carbonyl group C=O stretching bonds present in UiO-66(COOH)_2_. In the case of P-Z, an extra peak at 1590 cm^–1^ is related to C–N stretching vibrations.^37^ For P-A, the observed decrease in the intensity of the C–Cl stretching peak at around 1260 cm^–1^ suggests a strong interaction between silver cation and chlorine of the polymer (Figure S10).

**Figure 5:**
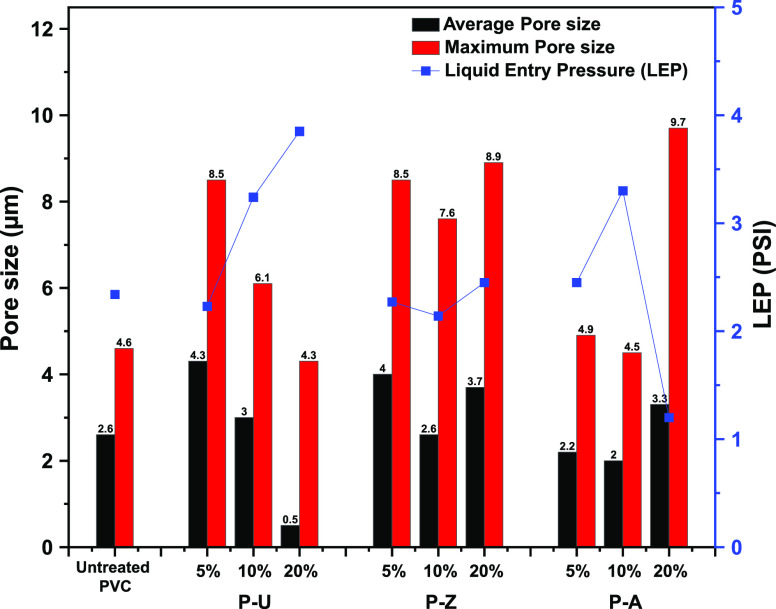
Relationship between pore size distribution and LEP for all treated membranes.

Water contact angle (WCA) and liquid entry pressure (LEP) are very important characteristics that give valuable insight into how the membrane is going to physically interact with polar molecules such as water. Table S2 shows the WCA measurements of all produced membranes in comparison with the untreated PVC. The results show that the obtained membranes are hydrophobic. Moreover, considering the standard deviation, we can conclude that after introducing MOFs-Ag or AgNO_3_, the WCA was not considerably affected, and the membranes kept their water-repelling characteristics. LEP is the measure of how much pressure the membrane can handle before failing and it is highly affected by hydrophobicity and the maximum pore size of the membrane.^38^ WCA measurements confirmed that the hydrophobicity was not compromised and, accordingly, the main contributor to the change of LEP was the pore size. Figure 5 summarizes the relation between the measured pore size and the LEP upon the addition of different MOFs. The LEP increased with the decrease in pore size for P-U and P-A membranes but slightly increased with the increase in pore size in the case of P-Z-treated membranes. Theoretically, the bigger pore size in P-Z membranes results in a lower LEP.^38^ However, due to the similarity in size between ZIF-8-Ag crystals and the average pore size of the membrane, “pore blockage” occurred, resulting in a reduction in the actual pore count (i.e., the number of pores present in the sample per unit area). This compensation for the high pore size distribution ultimately led to a comparable liquid entry pressure (LEP) to that of untreated PVC, which proves advantageous in our case as it enables high loading and relatively high LEP to be achieved.

For the MOFs-Ag-loaded membranes, the exact silver loading % was measured using AAS (see the Experimental Section for more details), while for P-A samples, TGA was employed, according to a protocol mentioned in the Supporting Information (SI). The results are shown in Table S3.

### Antibacterial Properties

As a proof of concept of the antibacterial activity of the prepared electrospun membranes, we tested the growth inhibition and the bactericidal effect of the prepared membranes against *E. coli* and *S. aureus* at 5, 10, and 20% MOFs-Ag loadings in P-U and P-Z systems. The percentage inhibition of *E. coli* was calculated relative to a positive control of 100% bacterial growth in the absence of any membrane after 18 h of incubation.

**Figure 6:**
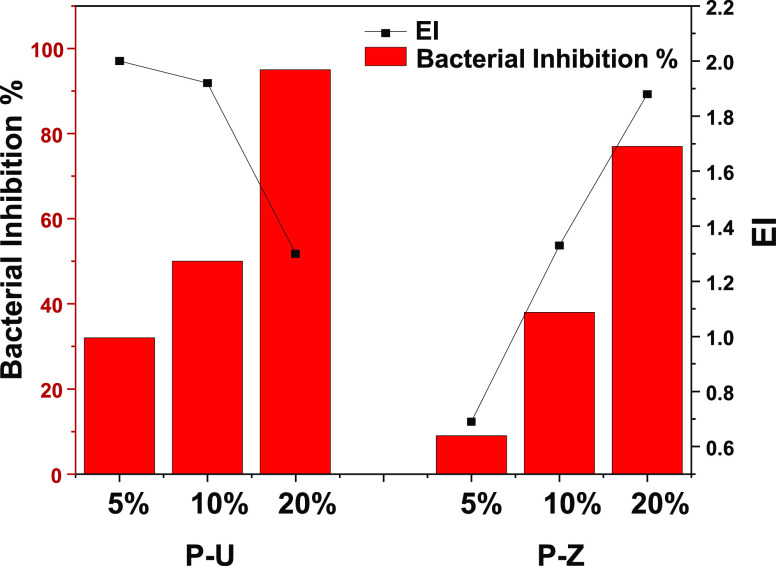
Relationship between bacterial inhibition (%) and EI for P-U and P-Z of different percentages by weight (5, 10, and 20%) at 15 mg/mL PVC/MOFs-Ag.

At 5% MOFs-Ag loading, the bacterial inhibition of P-Z was 9% with a calculated EI of 0.69, while that of P-U exhibited a much higher inhibition of 32% and an EI = 2 (Figure 6). When the MOFs-Ag loading percentage increased to 10%, both membranes showed an increase in bacterial inhibition to 38 and 50% for P-Z and P-U, respectively. However, when the effective inhibition was calculated, the P-U membranes showed no notable change with an EI of 1.92, while the P-Z effectiveness doubled to reach an EI value of 1.33 (Figure 6). A further increase in the MOFs-Ag loading to 20% led to an increase in inhibition to 77 and 95% for P-Z and P-U, respectively. The effective inhibition was calculated to be 1.88 for P-Z and 1.30 for P-U.

**Figure 7:**
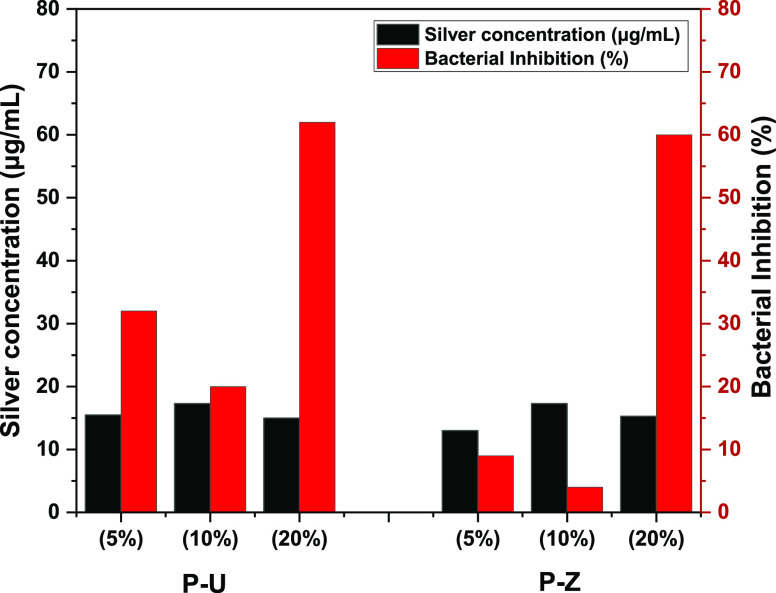
Difference in bacterial inhibition of P-U and P-Z membranes at 5, 10, and 20% MOFs-Ag loadings at the same silver concentration. The black-colored *y*-axis corresponds to the silver concentration, while the red-colored *y*-axis corresponds to the percentage of bacterial inhibition.

It is evident that the increase in surface coverage had a direct effect on the effective inhibition of the PVC/MOFs-Ag membranes. We, therefore, tested the bactericidal effect of PVC/MOFs-Ag membranes at different surface coverages but with similar silver concentrations. As seen in Figure 7, a substantial increase in bacterial inhibition is observed when the MOFs-Ag weight percent is increased to 20% in both systems. For instance, when the silver concentration is varied between 15 and 17 μg/mL and is tested for P-U at 5, 10, and 20%, the inhibition decreases from 32 to 20% and then increases to 62%, respectively. The same trend is observed with the P-Z at 5, 10, and 20% where the inhibition started at 9%, decreased to 4%, and then reached 60%, respectively.

We believe that this substantial enhancement in inhibition at 20 wt % is due to the increase in the local density of the MOFs-Ag particles, which supports a contact-based inhibition mechanism versus a slow Ag^+^ release. The higher local concentration might also inhibit the biofilm formation that creates an ideal environment for the bacteria for exponential growth and isolates the microorganism even under severe antibiotic treatment.^39^ While the increase in the antibacterial effect is expected with the increase in silver concentration following the MOFs-Ag loading percent, the effective inhibition factor enhancement is remarkable for the P-Z membranes (Figure 6). When looking at the changes in the physical properties of P-Z membranes compared to P-U, we find that P-Z exhibited a lower LEP overall (Figure 5). Therefore, it is easier for the microorganism to penetrate the P-Z membranes, which leads to better surface contact exposure with the metalated MOFs. This hypothesis is supported by the work of Regiel et al. who showed that the dispersion of silver nanoparticles on chitosan films affected the inhibition of biofilm-forming and antibiotic-resistant *S. aureus* after short contact times.^40^ The high biocidal effect is only achievable upon direct contact between the bacteria and the films and a little to no effect when the contact is eliminated. Direct contact inhibition is also demonstrated by Bondarenko et al. who not only showed the extracellular dissolution of silver but also the dissolution taking place at the particle–cell interface, which played an essential role in the antibacterial action of AgNPs with bacteria of the highest tendency to attach to nanoparticle surfaces exhibiting the highest sensitivity to all forms of nanoparticle Ag.^41^

**Figure 8:**
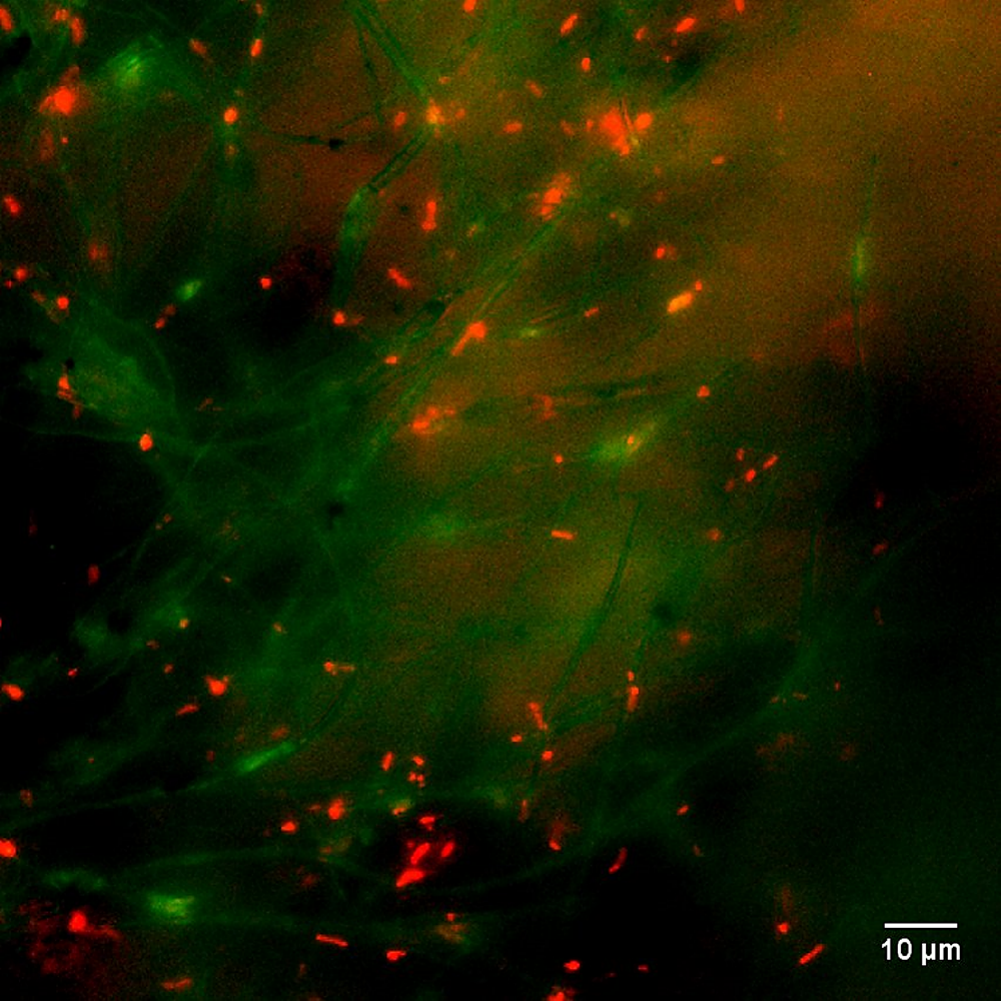
Fluorescence image of PVC/ZIF-8-Ag membranes with the LIVE/DEAD fluorescence assay and after 18–24 h of incubation with *E. coli*. Red-colored rods appear interspersed between the membrane nanofibers indicating dead bacteria.

To support our hypothesis of contact-based inhibition, LIVE/DEAD fluorescent microscopy was employed to observe bacterial cells on the membrane surface. Following an 18 h incubation of PVC/ZIF-8-Ag membranes with *E. coli* bacterial broth, the LIVE/DEAD fluorescence stain was added and incubated at room temperature for 15 min. Subsequently, fragments of the membrane were extracted and placed on glass slides for imaging using a fluorescent microscope at an excitation/emission of 480/500 nm for LIVE (green) and 490/635 nm for DEAD (red). The fluorescent images (Figure 8) reveal red-colored dead bacteria interspersed within the fibers of PVC/ZIF-8-Ag membranes when compared to the control (Figure S11), which shows no red-colored dead bacteria, thereby supporting our hypothesis of contact-based inhibition.

**Figure 9:**
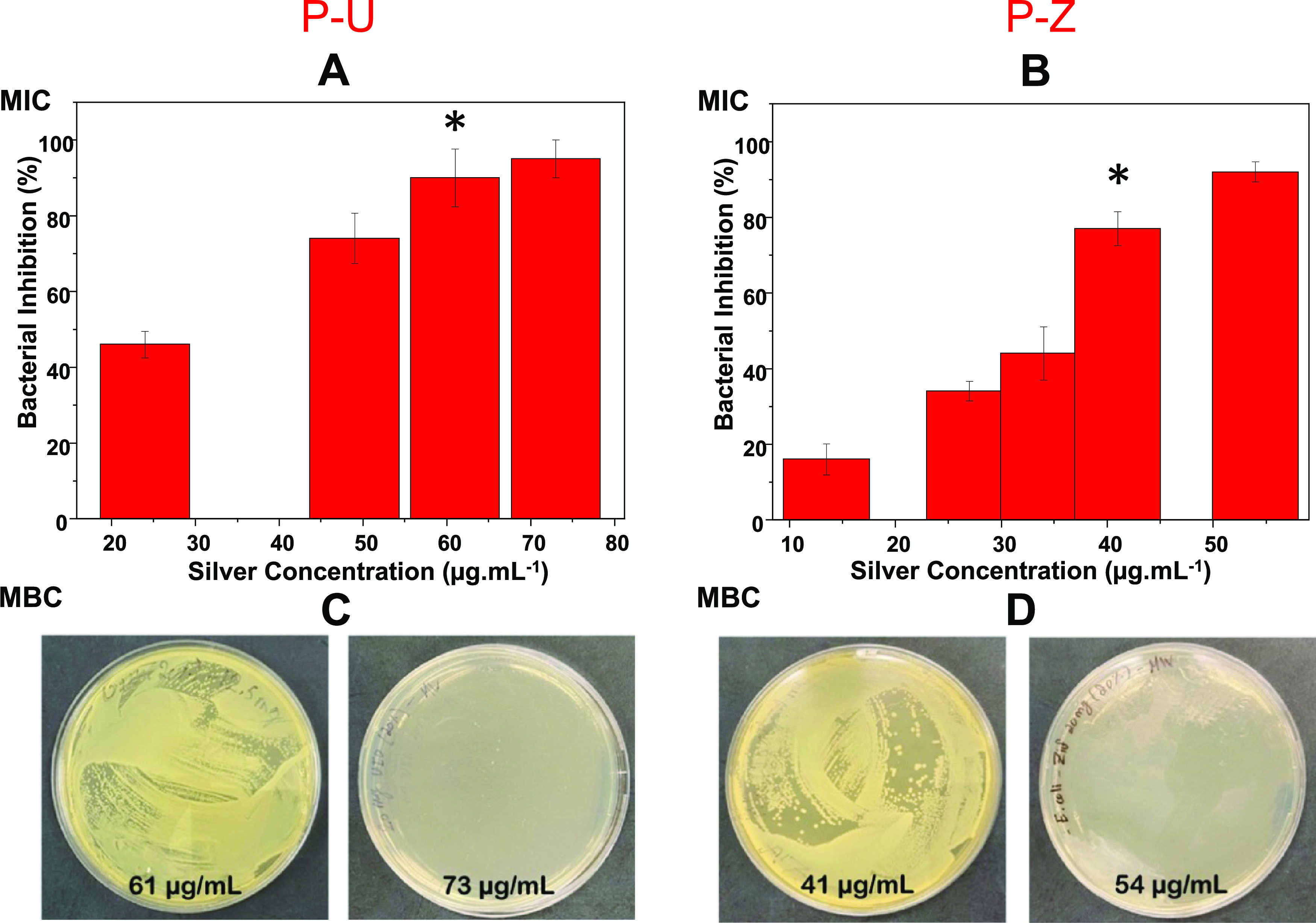
Graphs showing the MIC of P-U (A) and of P-Z (B) and MBC of P-U (C) and of P-Z (D) at 20% MOFs-Ag by weight of PVC against Gram-negative *E. coli.*

To calculate the MIC and MBC of the 20% modified PVC membranes, the antibacterial activity against *E. coli* was tested at a measured silver concentration between 7 and 97 μg/mL. The membranes were incubated overnight in freshly prepared bacterial cultures. The results were first assessed visually and then by measuring the optical density to determine the MIC. Serial dilutions from the incubated solutions are plated on agar and then kept at 37 °C for 18 h to determine the MBC. For the P-U membranes, the results showed antibacterial activity with an MIC of 61 μg/mL and an MBC of 73 μg/mL in calculated silver concentration. On the other hand, the P-Z membrane results showed slightly better antibacterial activity with an MIC of 41 μg/mL and an MBC of 54 μg/mL in calculated silver concentration (Figure 9). This observation could be the result of the difference observed in the physical characterization of the composite membranes (e.g., pore size and fiber diameter).

In a similar manner to the calculation of MIC and MBC values for the membranes against *E. coli*, the MIC and MBC for 20% P-U and P-Z membranes against *S. aureus* were determined using a silver concentration between 13.5 and 169 μg/mL. While it took a higher silver concentration to reach the MIC and MBC against *S. aureus* compared to *E. coli*, P-Z membranes still showed better antibacterial activity compared to P-U with an MIC of 70 μg/mL and an MBC of 95 μg/mL. P-U membranes, on the other hand, displayed lower antibacterial activity than P-Z membranes with an MIC of 126 μg/mL and an MBC of 169 μg/mL (Figure S12). This is a proof of concept that shows that our composite membranes have an effective bactericidal activity against both Gram-negative and Gram-positive bacteria.

The MICs and MBCs in this study are higher than the ones previously reported by our group for the freely suspended UiO-66(COOH)_2_-Ag (MIC and MBC of 6.5 μg/mL silver content). Nevertheless, this observation further supports the contact-based mechanisms that are more pronounced in a suspension solution. It also validates the use of MOFs-Ag in membranes for their potential applications in surgical masks and antibacterial films, which would not have been possible if the main mechanisms of antibacterial activities were based on the release of Ag cations.

To further validate the efficacy of the membranes, the antibacterial activity of PVC fibers prepared with silver nitrate P-A is assessed. At 5% AgNO_3_ by weight of PVC, we observed a 54% bacterial inhibition with an effective inhibition of 0.84. At 10%, P-A showed a bacterial inhibition of 91% with an effective inhibition of 0.40, and when increased to 20%, the MOF-based membranes showed a 95% inhibition and an EI of 0.24 (Figure S13). Moreover, P-A at 20% showed an MIC of 26 μg/mL and an MBC of 66 μg/mL (Figure S14). While the calculated MIC was better than those of P-Z and P-U, the amount of silver used in P-A was substantially much higher. Indeed, when the silver concentration was normalized, the bactericidal effect was more comparable with the reported EIs, confirming that P-Z and P-U exhibited a much higher effective inhibition than P-A.

Finally, a ZIF-8-Ag membrane with a significant size difference was prepared to further monitor the effect of the size on antibacterial activity. Our newly prepared PVC/ZIF-8-Ag membranes were prepared by a rapid synthesis method that uses DI water at room temperature.^42^ Then, the produced nanosized ZIF-8 MOFs were silver metalated using the same method^30^ of ZIF-8-Ag mentioned in the Experimental Section, resulting in composite membranes we call PVC/nZIF-8-Ag membranes. The results of the newly prepared nanoZIF-8-Ag (nZIF-8-Ag) are presented in Figure S15. According to the SEM images, the MOF has an average size of 100 nm, which is much smaller than the micro ZIF-8-Ag, which has an average size of 3 μm (Figure S15A). In the PVC/nZIF-8-Ag system, clusters also form as in the PVC/UiO-66(COOH)_2_-Ag system, which is logical given its size (Figure S15B). Following the same metalation protocol, the AAS results showed 1.34% silver in the samples, far less than the 8.1% silver in micro ZIF-8-Ag. As a result, micro-sized ZIF-8 had a better silver loading than nano-sized ZIF-8. This could be attributed to the defective surface of the nano-sized ZIF-8. As per the antibacterial results, Figure S15C demonstrates an obvious difference in the bacterial inhibition of nanosized nZIF-8-Ag in PVC/nZIF-8-Ag, which achieved a 13% inhibition, as compared with micro-sized ZIF-8-Ag in PVC/ZIF-8-Ag, which achieved a 38% inhibition at a 10% MOF loading. These results support our conclusion that MOF size plays an important role in bacterial inhibition within the nanofiber/MOF composite membrane system.

## Conclusions

In summary, the integration of MOFs, namely, UiO-66(COOH)_2_-Ag and ZIF-8-Ag, into PVC electrospun membranes led to high antibacterial activity that increased with MOFs-Ag % increase against both Gram-negative and Gram-positive bacteria. The antibacterial inhibition reached up to 95% with a 20% MOFs-Ag loading percentage. P-Z membranes demonstrated superior bacterial inhibition at similar MOFs-Ag loading percentages compared to P-U. This can be attributed to their larger pore sizes and bigger crystal structures. Furthermore, the MIC and MBC values of the 20% modified PVC membranes supported the utilization of PVC/MOFs-Ag in applications that require surface decontamination. Our results also revealed that the MOFs were evenly distributed within the PVC fibers, leading to alterations in the membranes’ morphology and color, as well as contributing to their thermal stability. Overall, the findings suggest that electrospun membranes containing MOFs-Ag serve as effective antibacterial materials, which could potentially be employed in applications such as medical devices, face masks, and even food packaging that necessitate surface decontamination.

## Supporting Information Available

Additional experimental details, membrane and MOF characterizations including SEM, TGA, FTIR, pore size analysis, and antibacterial tests (PDF)
