# Supplementary material for: Electrospun Metal–Organic Framework-Fabric Nanocomposites as Efficient Bactericides
Source: Langmuir. 2023 Jun 29;39(27):9503–13. doi: 10.1021/acs.langmuir.3c01039 (PMC10339785; doi:10.1021/acs.langmuir.3c01039)
Supplement: Supplementary file 1 — la3c01039_si_001.pdf [file la3c01039_si_001.pdf]

Supporting Information

Electrospun Metal Organic Framework-Fabric Nanocomposites as Efficient Bactericides

Mohammad H. Hashem[a]†, Mohamad Wehbe[b]†, Patrick Damacet[b], Rayan Kadah El Habbal[b], Nesreen Ghaddar[a], Kamel Ghali[a], Mohammad N. Ahmad[c], Pierre Karam[b]*, Mohamad Hmadeh[b]*.

a. Department of Mechanical Engineering, American University of Beirut, Beirut 1107 2020, Lebanon

b. Chemistry Department, American University of Beirut, P.O.Box 11-0236, Riad El-Solh, 1107 2020 Beirut, Lebanon

c. Bahaa and Walid Bassatne Department of Chemical Engineering and Advanced Energy, Faculty of Engineering and Architecture, American University of Beirut, P.O. Box 11-0236, Beirut 1107 2020, Lebanon

† Authors contributed equally

*Mohamad Hmadeh: mohamad.hmadeh@aub.edu.lb *Pierre Karam: pierre.karam@aub.edu.lb

Table of Content

Figure S1 Illustration of bacterial tests 2
Figure S2 Thermal Gravimetric analysis (TGA) of the non-metalated UiO-66(COOH)$_2$ (blue) and ZIF-8 (black) and metalated UiO-66(COOH)$_2$-Ag (orange), ZIF-8-Ag (green) MOFs. 3
Figure S3 FTIR spectrum of UiO-66(COOH)$_2$ (gray), UiO-66(COOH)$_2$-Ag (orange), ZIF-8 (black), ZIF-8-Ag (green) 3
Figure S4 Average fiber diameter of PVC, P-U (5, 10, 20 %), P-Z (5, 10, 20 %), P-A (5, 10, 20 %) membranes. 5
Figure S5 TGA for PVC/UiO-66(COOH)$_2$-Ag, P-U (5,10, 20 %) membranes. 5
Figure S6 TGA for PVC/ZIF-8-Ag P-Z (5, 10, 20 %) membranes. 6
Figure S7 SEM and optical images (1×1 cm) of PVC-$AgNO_3$ (P-A) membranes, (A1, A2) 5 %, (A3, A4) 10 %, (A5, A6) 20%. 6
Figure S8 TGA for PVC/$AgNO_3$, P-A (5, 10, 20 %) membranes. 7
Figure S9 Pore size distribution of the different membranes. 7
Figure S10 FTIR spectrum of PVC (balck), P-U (orange), P-Z (green) and P-A (wine) composite membranes 8

Figure S11 Fluorescence image of PVC with LIVE/DEAD Fluorescence as a control................8
Figure S12 Graphs showing the MIC of P-U (A) and of P-Z (B), and MBC of P-U (C) and of P-Z (D) at 20 % MOFs-Ag by weight of PVC against gram-positive S. aureus 9
Figure S13 The Relationship between bacterial inhibition and EI for P-A 9
Figure S14 Antibacterial inhibition of 20 % P-A membranes at MIC and MBC........................10
Figure S15 nZIF-8-Ag SEM micrographs (A), PVC/nZIF-8-Ag SEM micrographs (B), comparison in the bacterial inhibition at 10 % loading between PVC/nZIF-8-Ag and PVCZIF-8-Ag (C). 10

Table S1 Electrospinning Conditions used for PVC, P-U, P-Z and P-A:......................................2
Table S2 Water Contact Angle (WCA) of PVC, P-U (5, 10, 20 %), P-Z (5, 10, 20 %), P-A (5, 10, 20 %) membranes: 4
Table S3 Silver (Ag) loading percentage in the P-U, P-Z, P-A loaded samples: 4

Calculating the loading % in PVC/$AgNO_3$ (P-A) membranes: 11

Membranes and MOFs Characterization 12

Table S1 Electrospinning conditions used for PVC, P-U, P-Z and P-A:

Membrane	MOFs loading %	Voltage (KV)	Flow rate (ml/hr)	TCD	RPM
PVC	0	18	3	15	600
PVC/UiO-66$(COOH)_2$-Ag (P-U)	5	14	3	15	600
	10	14	3	15	600
	20	14	3	15	600
PVC/ZIF-8-Ag (P-Z)	5	18	3	15	600
	10	18	3	15	600
	20	18	3	15	600
PVC/$AgNO_3$ (P-A)	5	25	3	15	600
	10	25	3	15	600
	20	25	3	20	600

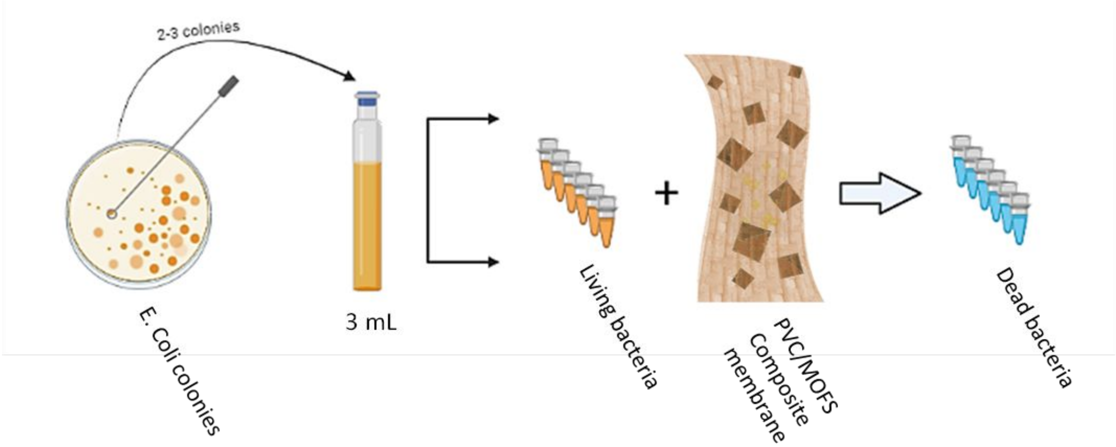

Figure S1 Illustration of bacterial tests

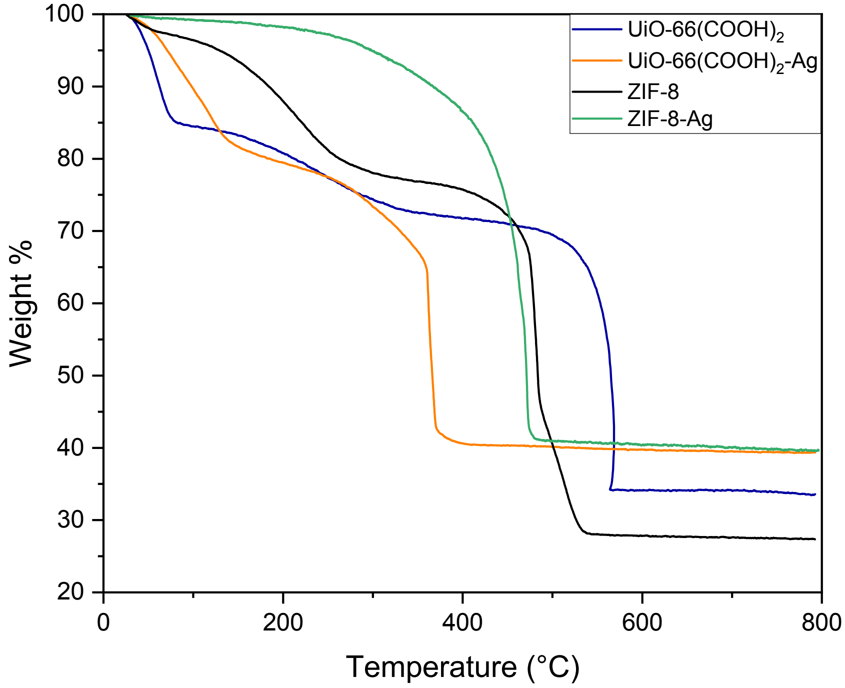

Figure S2 Thermal Gravimetric analysis (TGA) of the non-metalated UiO-66$(COOH)_2$ (blue) and ZIF-8 (black) and metalated UiO-66$(COOH)_2$-Ag (orange), ZIF-8-Ag (green) MOFs.

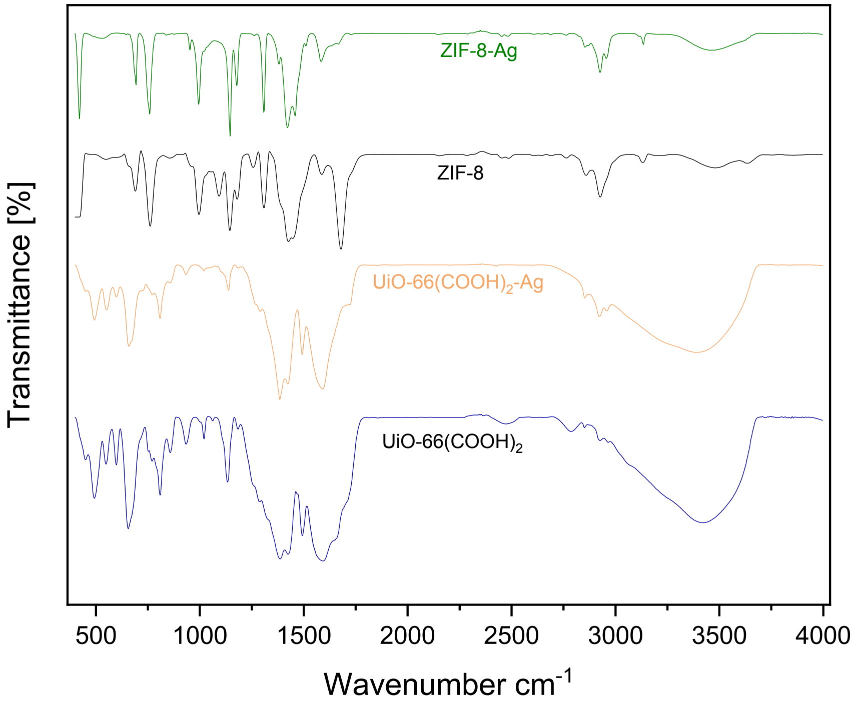

Figure S3 FTIR spectrum of UiO-66(COOH)$_2$ (gray), UiO-66(COOH)$_2$-Ag (orange), ZIF-8 (black), ZIF-8-Ag (green) .

Table S2 Water Contact Angle (WCA) of PVC, P-U (5, 10, 20 %), P-Z (5, 10, 20 %), P-A (5, 10, 20 %) membranes:

Membrane	**WCA °**	
**PVC**	135 ±2	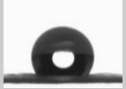
**P-U 5 %**	135 ±2	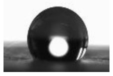
**P-U 10 %**	138 ±3	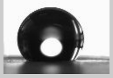
**P-U 20 %**	136 ±2	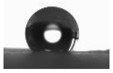
**P-Z 5 %**	133 ±2	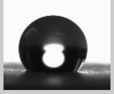
**P-Z 10 %**	133 ±2	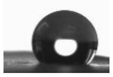
**P-Z 20 %**	128 ±3	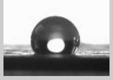
**P-A 5 %**	136 ±2	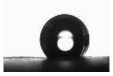

**P-A 10 %**	135 ±2	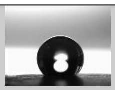
**P-A 20 %**	130 ±4	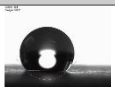

Table S3 Silver (Ag) loading percentage in the P-U, P-Z, P-A loaded samples:

Membrane	**Silver loading %**	**Method used**
**P-U 5 %**	0.31 %	(AAS)
**P-U 10 %**	0.52 %	(AAS)
**P-U 20 %**	1.46 %	(AAS)
**P-Z 5 %**	0.26 %	(AAS)
**P-Z 10 %**	0.57 %	(AAS)
**P-Z 20 %**	0.92 %	(AAS)
**P-A 5 %**	1.29 %	(TGA)
**P-A 10 %**	4.56 %	(TGA)
**P-A 20 %**	7.94 %	(TGA)

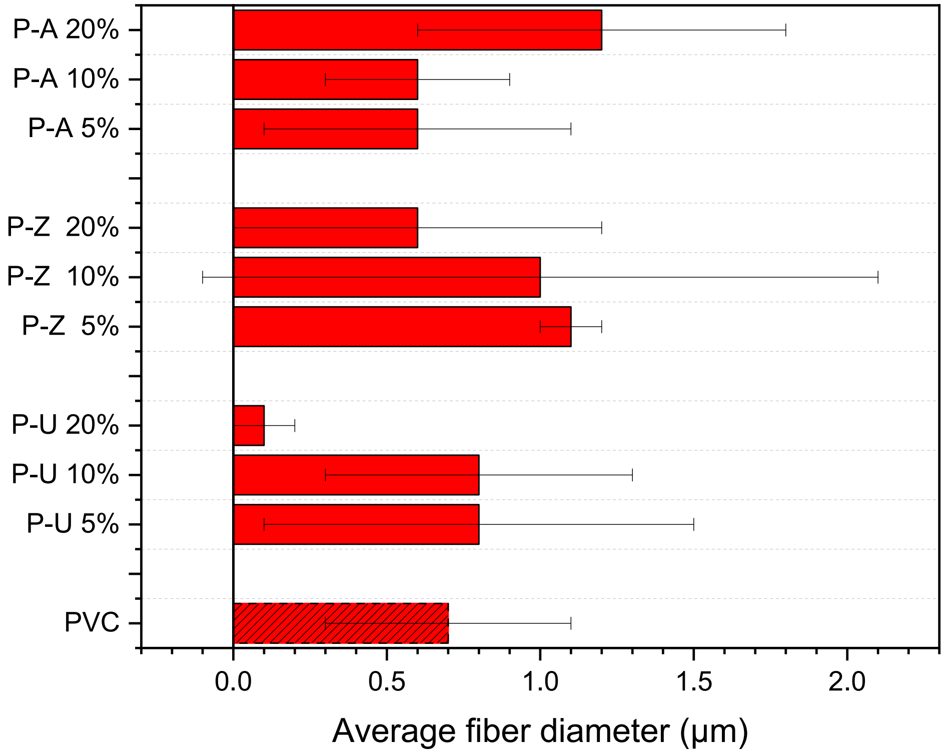

Figure S4 Average fiber diameter of PVC, P-U (5, 10, 20 %), P-Z (5, 10, 20 %), P-A (5, 10, 20 %) membranes.

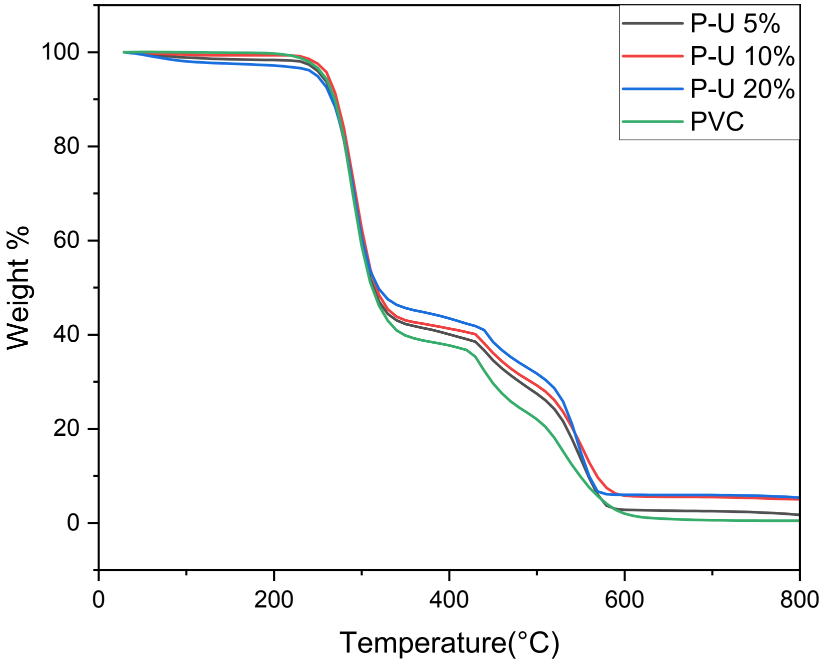

Figure S5 TGA for PVC/UiO-66$(COOH)_2$-Ag, P-U (5,10, 20 %) membranes.

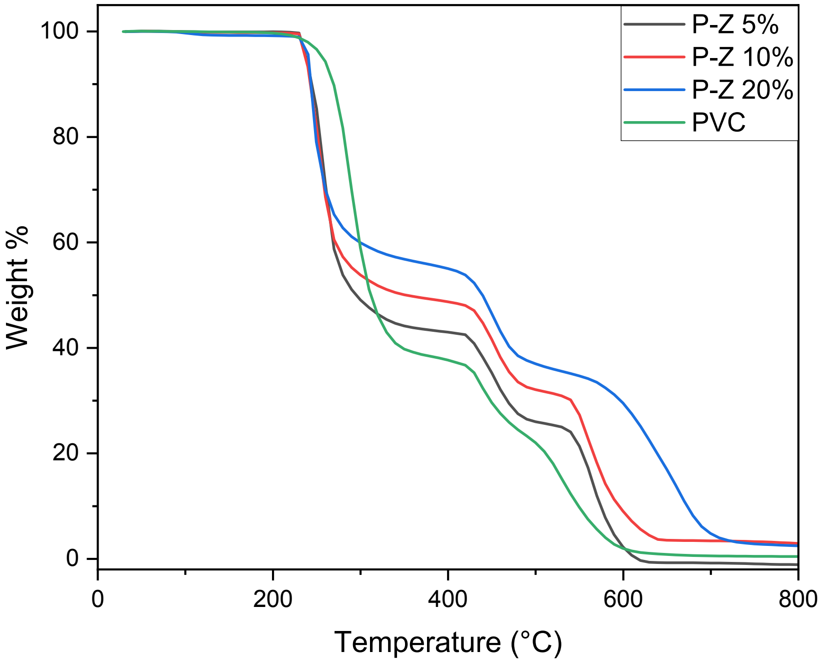

Figure S6 TGA for PVC/ZIF-8-Ag P-Z (5, 10, 20 %) membranes.

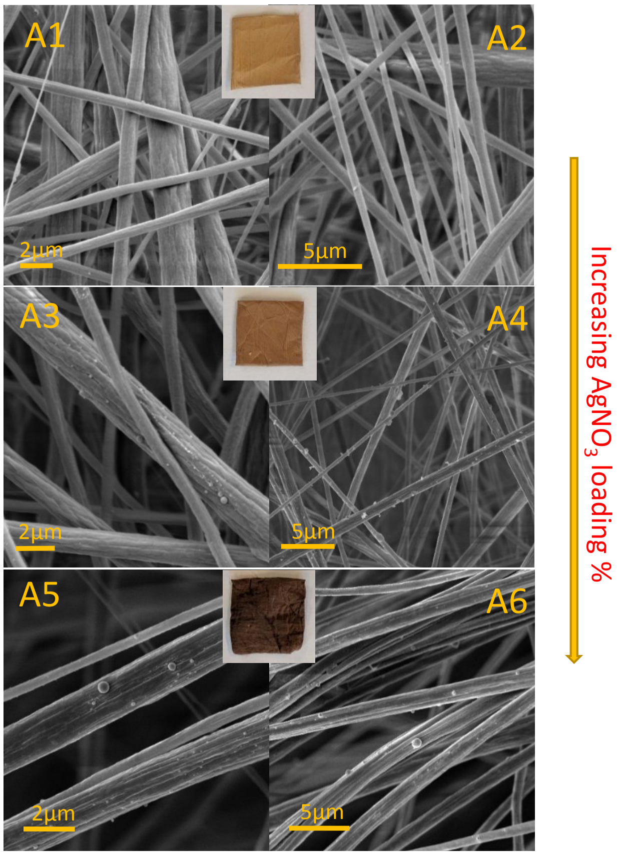

Figure S7 SEM and optical images (1×1 cm) of PVC-$AgNO_3$ (P-A) membranes, (A1, A2) 5 %, (A3, A4) 10 %, (A5, A6) 20%.

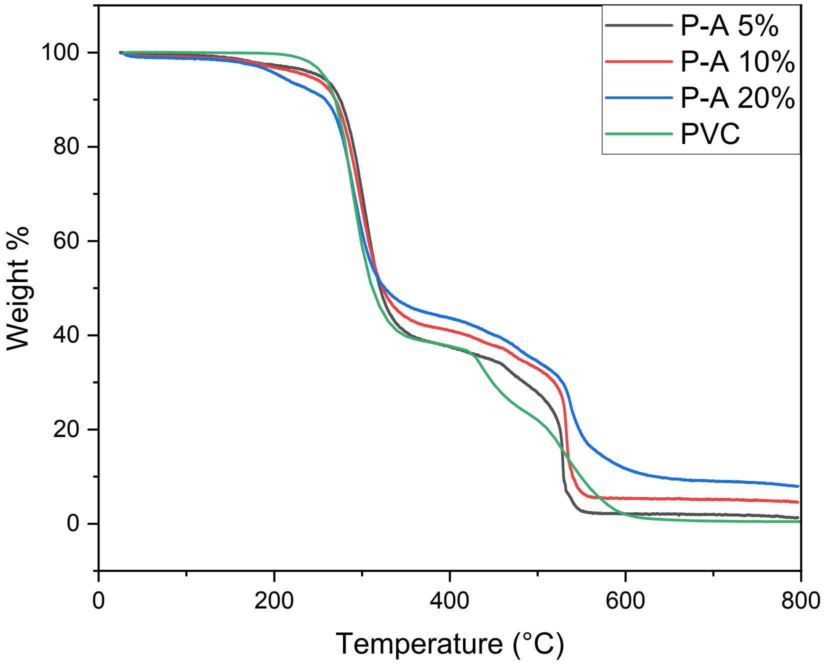

Figure S8 TGA for PVC/$AgNO_3$, P-A (5, 10, 20 %) membranes.

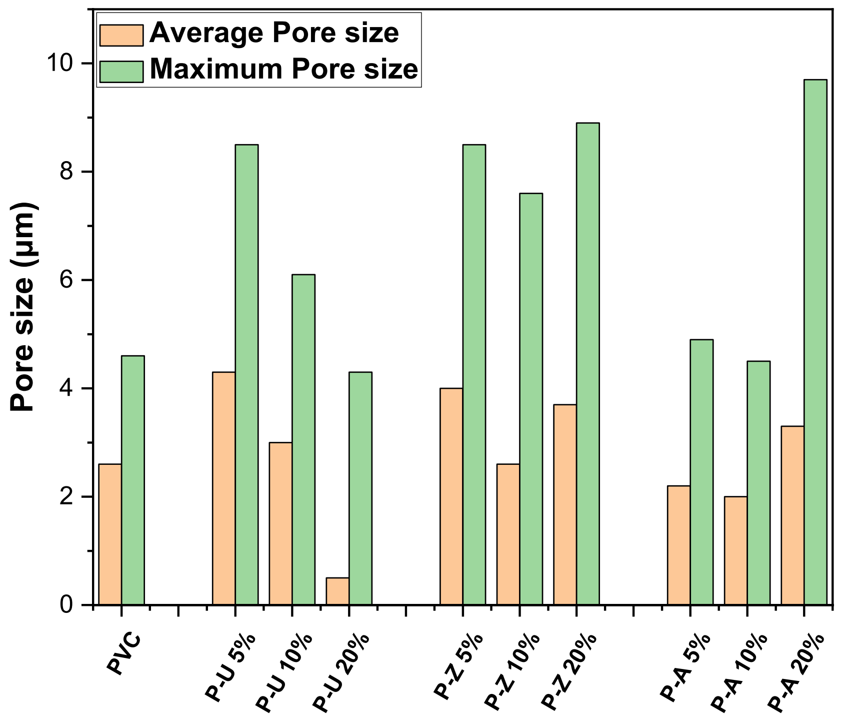

Figure S9 Pore size distribution of the different membranes.

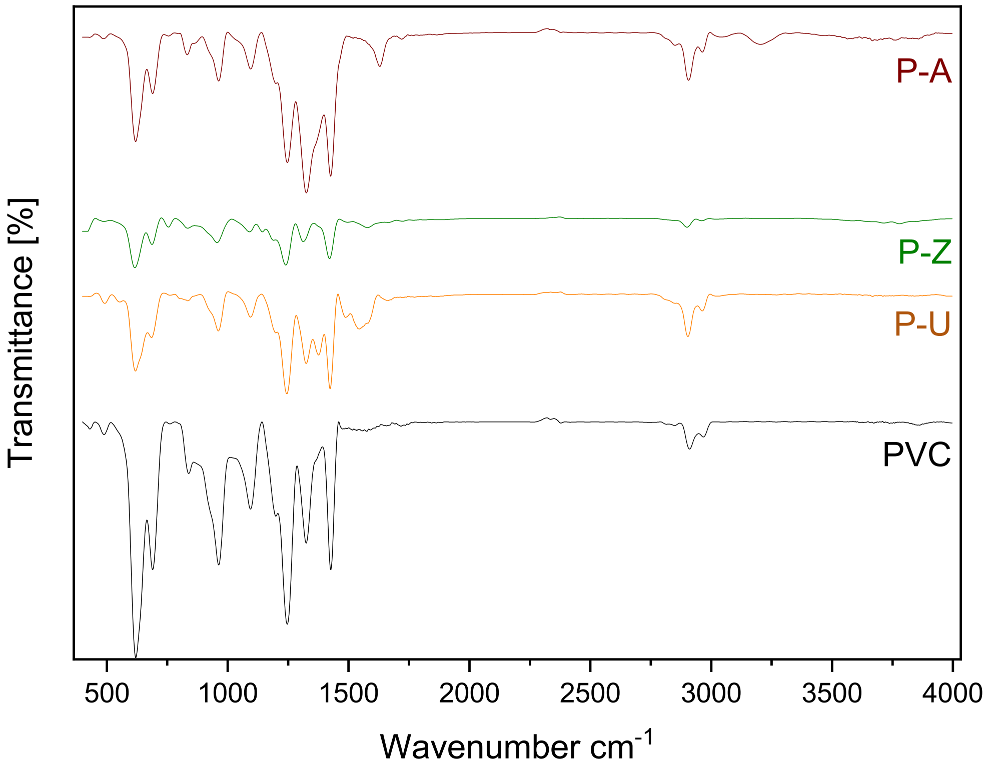

Figure S10 FTIR spectrum of PVC (balck), P-U (orange), P-Z (green) and P-A (wine) composite membranes

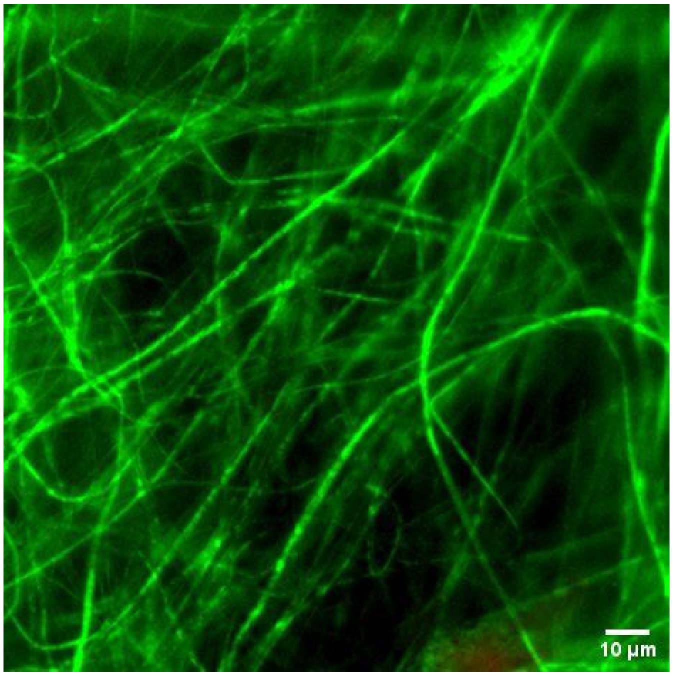

Figure S11 Fluorescence image of PVC with LIVE/DEAD Fluorescence as a control

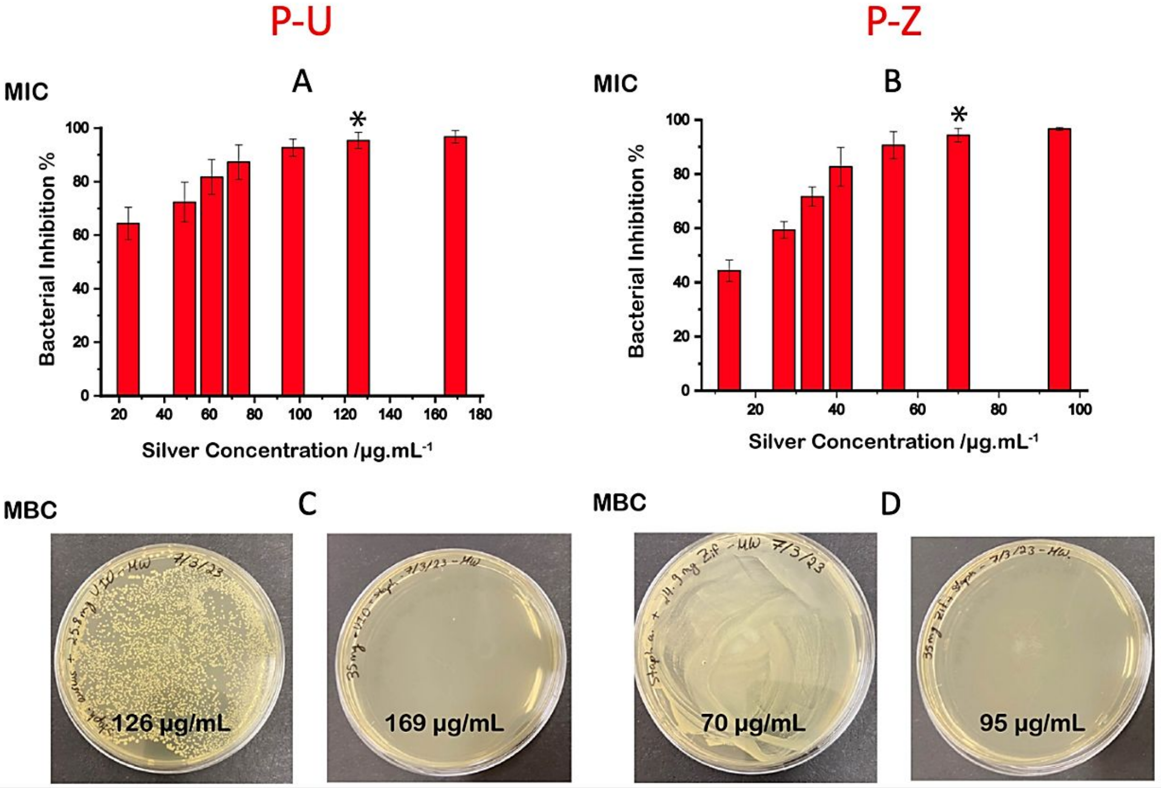

Figure S12 Graphs showing the MIC of P-U (A) and of P-Z (B), and MBC of P-U (C) and of P-Z (D) at 20 % MOFs-Ag by weight of PVC against gram-positive S. aureus

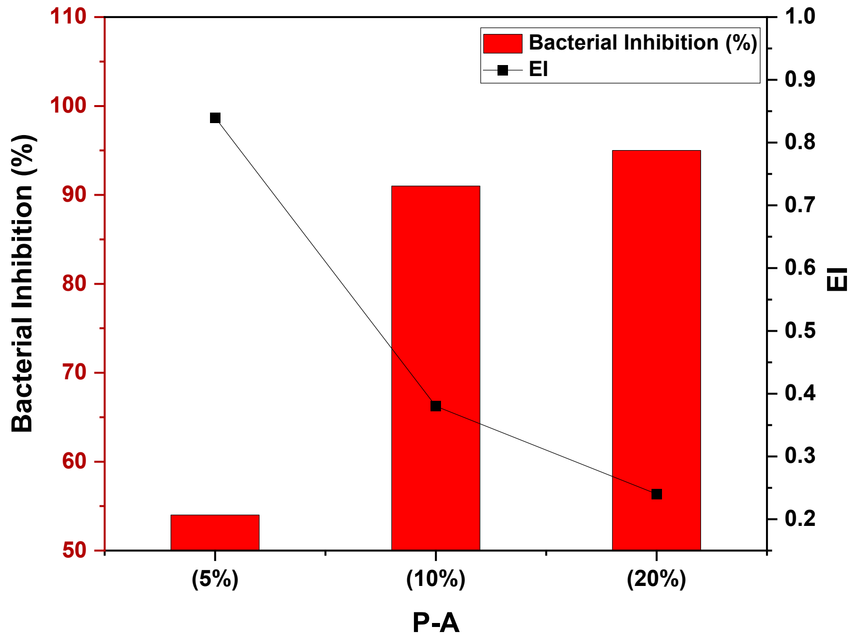

Figure S13 The Relationship between bacterial inhibition and EI for P-A

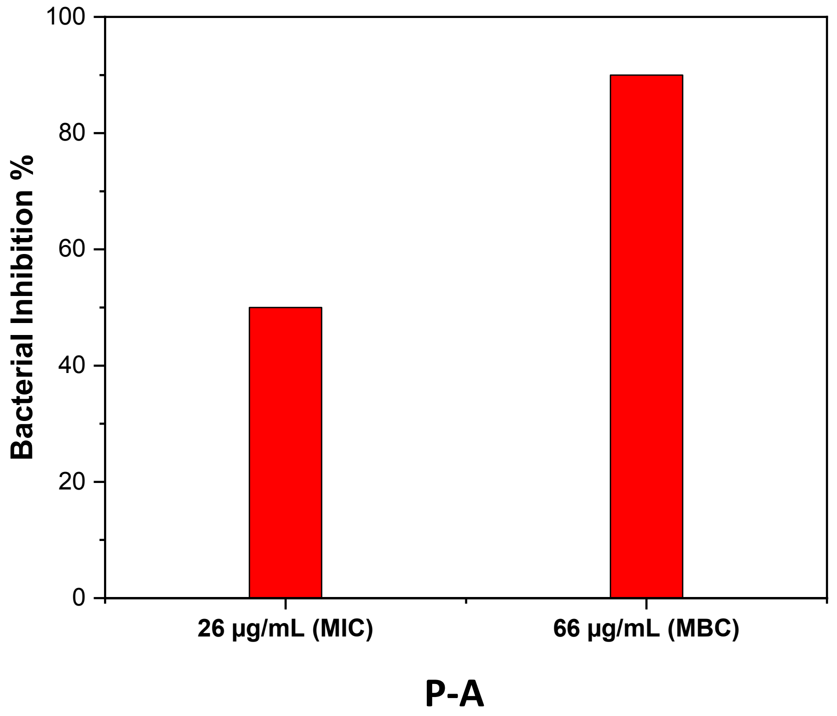

Figure S14 Antibacterial inhibition of 20 % P-A membranes at MIC and MBC

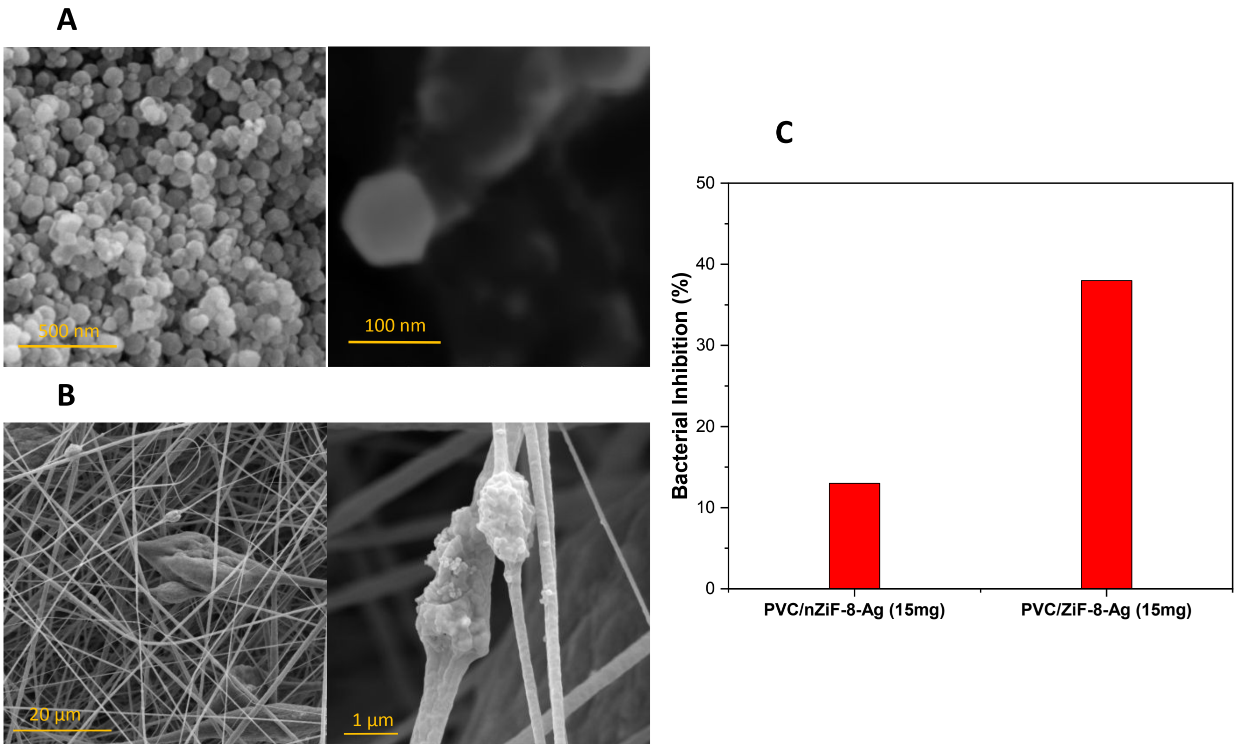

Figure S15 nZIF-8-Ag SEM micrographs (A), PVC/nZIF-8-Ag SEM micrographs (B), comparison in the bacterial inhibition at 10 % loading between PVC/nZIF-8-Ag and PVCZIF-8-Ag (C).

Calculating the loading % in PVC/$AgNO_3$ (P-A) membranes:

At high temperatures T > 440 °C, $AgNO_3$ will decompose to give silver metal and other gases, this will allow us to calculate the Ag % loading in PVC/$AgNO_3$ samples the decomposition equation is shown below:

$$2AgNO_3 \rightarrow 2Ag + 2NO_2 + O_2 \tag{S1}$$

From the Thermal Gravimetric graph, the remaining weight of the (5, 10 and 20 %) samples are **1.29, 4.56 and 7.94 %** respectively. And as mentioned before, according to the equation, this represents the Ag % loading in the corresponding samples.

Membranes and MOFs Characterization

A thickness gauge (MP-1, Brunswick Instrument, and UK) with a probe tip and floating stand anvil were used for measuring the thickness of produced membranes. The measurements were collected according to ASTM-D1777 by applying a pressure of (~1464 Pa) on the objects.

The membranes' morphology was assessed by scanning electron microscopy technique (SEM MIRA 3 LMU Tescan, Czech Republic) with an In-Beam detector. Before SEM measurements, samples were gold coated with a thin layer (10 nm) using a (Q150 T) turbo-pumped sputter coater (Quorum Technologies).

The water contact angle formed on the hydrophobic porous electrospun membranes surface was measured using an optical tensiometer (OCA 15EC, Data Physics, Germany). The droplets ranged between 1 and 5 μL. The contact angle is defined as the angle formed between the liquid-vapor interface and the liquid-solid interface.

A Capillary Flow Porometer (CFP-1100AH, PMI, NY, USA) instrument was used to estimate the pore size distribution within the fabricated membranes. Briefly, a membrane was inserted into the membrane cell holder using Galwick (of low surface tension 15.9 mN/m) as the wetting agent. The flow of saturated air (Q), passing through the membrane was steadily increased, and the resultant pressure difference (ΔP) was measured. The pore size distribution was then calculated based on the measured flow rate (Q) as a function of pressure difference (ΔP).

The Liquid Entry Pressure (LEP) of the hydrophobic porous membranes was measured using the CFP. For this measurement, a layer of DI water was added on top of the membrane to measure the maximum hydrostatic pressure that a membrane can tolerate before failing (leaking).

For the membrane thermal analysis, a Thermo-Gravimetric analysis (TGA) Q500 was used. The temperature range was set between 30 °C and 1000 °C with a step of 10 °C□/min. Powder X-ray diffraction (PXRD) patterns were collected using a Bruker D8 advance X-ray diffractometer (Bruker AXS GmbH, Karlsruhe, Germany).

For monitoring the functionality of the samples Fourier-transform infrared spectroscopy (FTIR) (TG-FTIR (TGA-FTIR) Thermogravimetric Analysis by Bruker Optics) with using the KBr wafer technique was utilized. In addition, X-ray photoelectron spectroscopy (XPS) was used to analyze the surface composition.

The amount of silver incorporated in the frameworks was determined by iCE 3000 series Atomic Absorption Spectrophotometer (AAS) using air-acetylene as a fuel source. Briefly, 0.6 mg of each composite was transferred to a falcon tube containing 200 µL of an Aqua Regia solution. For complete dissolution, the mixture was then sonicated for a few minutes followed by the addition of 200 µL of hydrofluoric acid (HF, 50 %). Finally, 10 mL of deionized water was added for dilution. Silver weight % was calculated using the below formula:

$$\% \, Ag \, (Weight) = \frac{C \, x \, V}{m} \times 100 \qquad \text{(S2)}$$

Where *C* is the silver mass concentration in the MOFs sample in ppm (mg/L) determined via AAS, *V* is the total volume of the solution in L, and *m* is the mass of the MOFs sample used in mg. Silver concentrations were determined by multiplying the calculated silver % by the weight of the membrane used in each sample to be converted into a concentration of µg/mL.
